# Structural and Biochemical Bases for the Redox Sensitivity of *Mycobacterium tuberculosis* RslA

**DOI:** 10.1016/j.jmb.2010.02.026

**Published:** 2010-04-16

**Authors:** Krishan Gopal Thakur, T. Praveena, B. Gopal

**Affiliations:** Molecular Biophysics Unit, Indian Institute of Science, Bangalore 560 012, India

**Keywords:** RNAP, RNA polymerase, ASD, anti-σ domain, ECF, extracytoplasmic function, *Mtb*, *Mycobacterium tuberculosis*, *Ec*, *Escherichia coli*, *Sco*, *Streptomyces coelicolor*, ZAS, zinc-associated anti-σ, *Rsp*, *Rhodobacter sphaeroides*, PDB, Protein Data Bank, PAR, 4-(2-pyridylazo)-resorcinol, SPR, surface plasmon resonance, LC-ESI-MS, liquid chromatography–electrospray ionization–mass spectrometry, MALDI-TOF, matrix-assisted laser desorption/ionization time-of-flight, wt, wild type, DLS, dynamic light scattering, TPEN, *N*,*N*,*N*′,*N*′-tetrakis(2-pyridylmethyl) ethylenediamine, extracytoplasmic function σ factor, zinc binding, redox sensitivity, anti-σ factor

## Abstract

An effective transcriptional response to redox stimuli is of particular importance for *Mycobacterium tuberculosis*, as it adapts to the environment of host alveoli and macrophages. The *M. tuberculosis* σ factor σ^L^ regulates the expression of genes involved in cell-wall and polyketide syntheses. σ^L^ interacts with the cytosolic anti-σ domain of a membrane-associated protein, RslA. Here we demonstrate that RslA binds Zn^2+^ and can sequester σ^L^ in a reducing environment. In response to an oxidative stimulus, proximal cysteines in the CXXC motif of RslA form a disulfide bond, releasing bound Zn^2+^. This results in a substantial rearrangement of the σ^L^/RslA complex, leading to an 8-fold decrease in the affinity of RslA for σ^L^. The crystal structure of the − 35-element recognition domain of σ^L^, σ_4_^L^, bound to RslA reveals that RslA inactivates σ^L^ by sterically occluding promoter DNA and RNA polymerase binding sites. The crystal structure further reveals that the cysteine residues that coordinate Zn^2+^ in RslA are solvent exposed in the complex, thus providing a structural basis for the redox sensitivity of RslA. The biophysical parameters of σ^L^/RslA interactions provide a template for understanding how variations in the rate of Zn^2+^ release and associated conformational changes could regulate the activity of a Zn^2+^-associated anti-σ factor.

## Introduction

The presence of multiple σ factors and the conditional association of these proteins with RNA polymerase (RNAP) are essential components of the prokaryotic transcriptional machinery that is sensitive to a diverse range of developmental and environmental stimuli. The activity of σ factors is often regulated by their association with an anti-σ factor.^1,2^ Despite poor sequence similarity, the structures of both σ factors, as well as the anti-σ domains (ASDs) of the anti-σ factors, are remarkably well conserved.^3–6^ σ factors belonging to the extracytoplasmic function (ECF) family often govern changes in transcription in response to environmental stimuli.^7,8^ Of the 13 σ factors in *Mycobacterium tuberculosis* (*Mtb*), 10 belong to the ECF family. A characteristic feature of this bacillus is the long period of dormancy in the host, which is also referred to as the latent phase of mycobacterial growth. It is therefore not surprising that several ECF σ factors are involved in mediating a transcriptional response to oxidative stimuli, in addition to the primary σ factors. The oxidative stress response that partially overlaps with the disulfide response mechanism is remarkably diverse in bacteria, involving chaperones such as the *Escherichia coli* (*Ec*) chaperone Hsp33 or receptor proteins such as the anti-σ factor RsrA from *Streptomyces coelicolor* (*Sco*).^9–11^ Since the characterization of *Sco* RsrA, several anti-σ factors have been noted to have a conserved HXXXCXXC motif, a finding that provided a basis for a class of proteins called the zinc-associated anti-σ (ZAS) factor. The differences between two well-characterized ZAS proteins, *Sco* RsrA and *Rhodobacter sphaeroides* (*Rsp*) ChrR, however suggested that the mechanism by which these ZAS proteins detect oxidative signals and release the bound σ factor is quite diverse. In the case of the *Sco* σ^R^/RsrA system, Zn^2+^ binding and σ^R^ association are coupled events; Zn^2+^ release, in tandem with the formation of multiple disulfide bonds, was noted to be a critical feature of the RsrA sensor.^12,13^ Detection of singlet oxygen by *Rsp* ChrR, on the other hand, was shown to be localized to a cupin domain and not to the ASD that inactivates σ^E^.^3^

A search for ZAS homologues in *Mtb* suggested that three anti-σ factors regulating the ECF σ factors σ^E^, σ^H^, and σ^L^ have the HXXXCXXC motif.^11^ σ^E^ and σ^H^ respond to oxidative stress *in vivo*.^14,15^ With *in vitro* transcription assays, the anti-σ factor of σ^L^, RslA, was shown to inhibit σ^L^-dependent transcription of the σ^B^ gene in a dose-dependent manner.^16^ It was further noted that many of the genes regulated by σ^L^ are involved in processes related to the cell envelope or secreted protein modifications.^16,17^ Here we describe the structural and biochemical features of *Mtb* RslA and its interactions with σ^L^. We demonstrate that the ASD of RslA binds Zn^2+^ and responds to oxidative stress by forming a disulfide bond between the proximal cysteine residues of the CXXC motif. We note that Zn^2+^ release under oxidizing conditions is a rapid response, while the consequent structural changes in RslA are much slower. Of the two promoter recognition domains in σ^L^, σ_4_^L^ interacts with RslA with a higher affinity when compared to σ_2_^L^. The crystal structure shows that the orientation of domains in the σ_4_^L^/RslA complex is different from that in *Rsp* ChrR, the only other structurally characterized ZAS–σ factor complex. Unlike *Rsp* ChrR, no structural rearrangements were seen in σ_4_^L^ upon RslA binding. The Zn^2+^ binding and biochemical features of RslA mutants suggest that surface accessibility of the conserved CXXC motif plays a more important role in the redox sensitivity of a ZAS protein than the XX dipeptide sequence between the cysteine residues. The structure of the σ_4_^L^/RslA complex thus provides a basis for understanding variations in the redox sensitivity of ZAS proteins.

## Results

### The crystal structure of the σ_4_^L^/RslA complex

**Supplementary Fig. 1:**
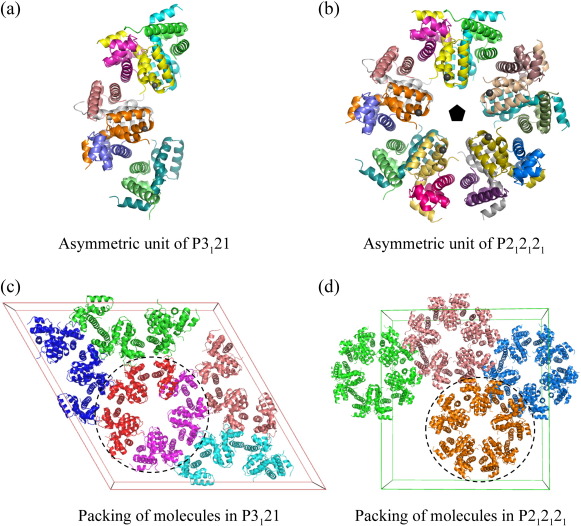
(a and b) Arrangement of σ_4_^L^/RslA complex molecules in an asymmetric unit with *P*3_1_21 and *P*2_1_2_1_2_1_ crystal forms. All chains are colored differently. Zinc atoms are shown as gray spheres. (c and d) Packing of the σ_4_^L^/RslA complex molecules in two different crystal forms. Five molecules of the σ_4_^L^/RslA complex in the asymmetric unit of the *P*3_1_21 crystal form, along with 2-fold crystallographic symmetry, result in a similar packing arrangement as seen in the *P*2_1_2_1_2_1_ crystal form with 10 molecules of the σ_4_^L^/RslA complex molecules in an asymmetric unit (dotted circles).

**Fig. 1:**
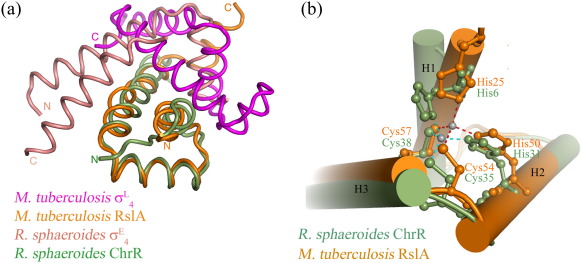
The crystal structure of the − 35-element recognition domain of σ^L^, σ_4_^L^, bound to RslA. (a) The interaction of the ASD of *Mtb* RslA with σ_4_^L^ when compared with the *Rsp* σ_4_^E^/ChrR complex.^3^ While the ASDs of *Mtb* RslA (orange) and *Rsp* ChrR (green) superpose well, those of *Mtb* σ_4_^L^ (magenta) and *Rsp* σ_4_^E^ (salmon) do not. (b) Zn^2+^ coordination in *Mtb* RslA and Zn^2+^ coordination in *Rsp* ChrR are identical.

Crystals of the σ^L^/RslA complex diffracted to 3.2 Å, had high mosaicity, and took several months to obtain. These crystals belonged to space group *P*3_1_21 with unit cell parameters *a* = 167.46 Å, *b* = 167.46 Å, and *c* = 64.65 Å. Subsequently, we noted that these crystals contained only the σ_4_^L^ domain and RslA; σ_2_^L^ appeared to have proteolyzed in the course of crystallization. Crystals were readily obtained from an expression construct that had σ_4_^L^ and RslA. These crystals diffracted to 2.4 Å resolution, and the structure of the σ_4_^L^/RslA complex was solved using single-wavelength anomalous dispersion, with data collected at the Zn^2+^ K-edge [Protein Data Bank (PDB) ID 3HUG]. Data collection and refinement statistics are summarized in Supplementary Table 1. There are 10 molecules of the σ_4_^L^/RslA complex in the asymmetric unit. In this arrangement, five heterotetramers of the σ_4_^L^/RslA complex pack with a 5-fold symmetry to form a ring-like structure, with the zinc binding region of RslA facing the center of the ring. This arrangement results in a large pore with a diameter of about ∼ 25 Å. A pictorial representation of the packing of the σ_4_^L^/RslA complex molecules in the two crystal forms is shown in Supplementary Fig. 1. The 1:1 stoichiometry for the σ_4_^L^/RslA complex seen in the crystal structure is consistent with biochemical data (Fig. 1a). About 23 residues in the N-terminus and 22 residues in the C-terminus were disordered in the crystal structure of RslA. σ_4_^L^ forms a tight complex with RslA involving a buried surface area of ca 1332 Å^2^. The Zn^2+^ cofactor in RslA is coordinated by His25, His50, Cys54, and Cys57 (Fig. 1b). Thus, all the conserved residues in the HXXXCXXC motif are involved in Zn^2+^ binding.

**Supplementary Fig. 2:**
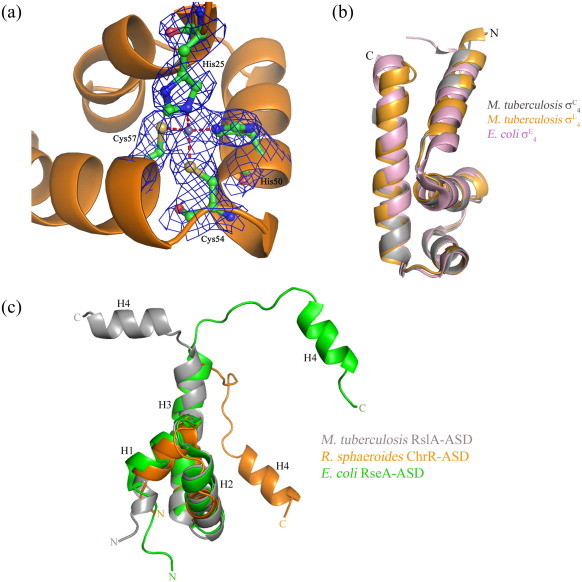
(a) An (*mF*_o_ − *DF*_c_) electron density map showing the coordination of Zn^2+^ in the structure of the σ_4_^L^/RslA complex. (b) Binding of RslA does not induce structural changes in σ_4_^L^. A structural superposition of the crystal structures of region 4 corresponding to ECF σ domains is shown here. (c) Helix H4 of ASDs adopts different conformations, while the other three helices superpose well.

**Supplementary Fig. 3:**
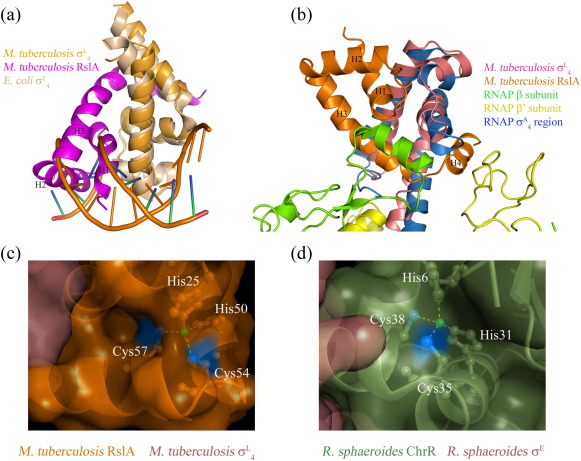
(a) RslA sterically occludes the promoter recognition region of σ_4_^L^. The σ_4_^L^/RslA complex was superposed on the crystal structure of the σ_4_^E^/− 35 promoter DNA complex (PDB ID 2H27).^20^ (b) Docking of the σ_4_^L^/RslA complex onto *Thermus thermophilus* holo-RNAP (PDB ID 1IW7).^22^ Docking of RslA on polymerase reveals several steric clashes of RslA with a helical stretch of the β-subunit. (c) Surface representation of the region containing bound Zn^2+^ in the ASDs of RslA and ChrR. The sulfur atoms of the cysteine residues (Zn^2+^ binding) are shown in blue. Both cysteines in the CXXC motif in *Mtb* RslA are accessible to the solvent. The solvent-accessible cysteines in *Mtb* RslA provide a structural rationale for the redox sensitivity of this ASD. (d) A surface representation of *Rsp* ChrR in a similar orientation as *Mtb* RslA in (c). The Cys38 of the CXXC motif is occluded by Arg41 (*Rsp* ChrR numbering).

The structure of the σ_4_^L^ domain superimposes well with those of *Mtb* σ_4_^C^ (PDB ID 2O8X; RMSD of 0.74 Å over 60 C^α^)^6^ and *Ec* σ_4_^E^ (PDB ID 1OR7; RMSD of 1.64 Å over 68 C^α^)^4^ (Supplementary Fig. 2). Thus, RslA binding does not lead to a structural rearrangement of σ_4_^L^, unlike the case of the *Rsp* σ^E^/ChrR complex (PDB ID 2Z2S) or the *Ec* σ_4_^E^/AsiA complex (PDB ID 1TLH).^3,18^ Superposition of the structure of RslA on that of the *Rsp* σ^E^/ChrR complex shows that only three helices of the four-helical domain of RslA overlap on *Rsp* ChrR. Helix H4 adopts a different orientation (Supplementary Fig. 2c). This helix (H4) occludes the RNAP β′ coiled-coil binding surface of a σ factor.^3,19^ The orientation of the σ_4_ domains in the σ_4_^L^/RslA complex is different from that seen in the *Rsp* σ^E^/ChrR complex (Fig. 1). A possible inhibitory mechanism of RslA can be inferred from the superposition of the σ_4_^L^/RslA structure on that of the σ_4_^E^/− 35 promoter DNA complex (PDB ID 2H27).^20^ This superposition revealed several steric clashes with the DNA. Modeling of the σ_4_^L^/RslA complex on RNAP (PDB ID 1IW7) also suggested steric clashes of RslA with the β flap–tip–helix of RNAP^21,22^ (Supplementary Fig. 3a and b). RslA is thus likely to inactivate σ^L^ by occluding both the promoter and the RNAP binding regions of σ_4_^L^ without any structural rearrangements.

### Zn^2+^ release precedes conformational changes in RslA with an oxidative stimulus

To evaluate the role of bound Zn^2+^ in RslA, we characterized the kinetics of Zn^2+^ release and conformational changes in RslA with an oxidative stimulus. Several expression constructs were examined to obtain a σ^L^/RslA complex that could be purified and concentrated without aggregation or precipitation. A list of expression constructs examined in this study is compiled in Supplementary Table 2. All the biophysical and biochemical studies reported here were performed on full-length σ^L^ (residues 1–177) and on the ASD of RslA (residues 1–108).

**Fig. 2:**
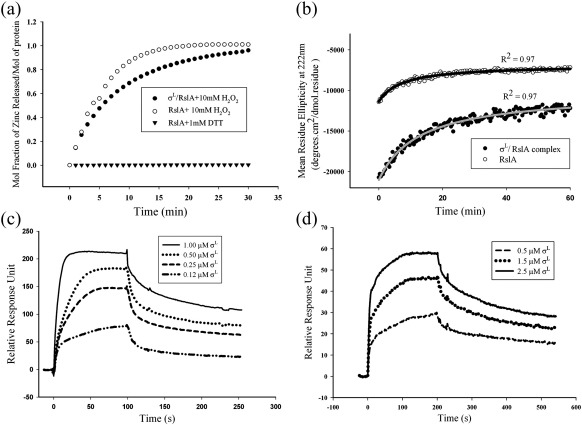
RslA binds Zn^2+^ under reducing conditions. (a) Zn^2+^ release monitored using the chromophore PAR. (b) Time-course measurements of structural changes in RslA and in the σ^L^/RslA complex monitored by CD. (c) SPR measurements of σ^L^/RslA interactions. RslA forms a complex with σ^L^ with a *K*_d_ of ca 20 nM under reducing conditions. (d) Interactions of apo-RslA with σ^L^. An 8-fold decrease in binding, corresponding to a *K*_d_ of ∼ 150 nM, was observed.

**Fig. 3:**
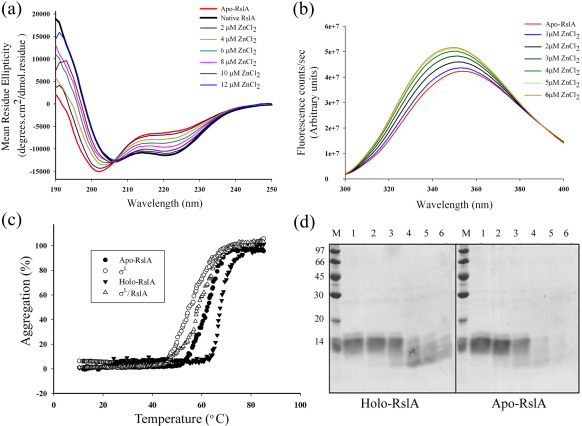
Zinc removal with an oxidative stimulus induces conformational changes in RslA and in the σ^L^/RslA complex. (a) CD spectra showing changes in the secondary structure of RslA with increasing concentration of Zn^2+^ under reducing conditions. (b) Zinc binding monitored by intrinsic tryptophan fluorescence. Zinc binding stabilizes helix H1 of RslA. (c) Zinc binding confers stability to RslA and the σ^L^/RslA complex. Temperature-induced denaturation of σ^L^, RslA, and the σ^L^/RslA complex. As seen by thermal melting profiles, Zn^2+^ binding confers stability to RslA and the σ^L^/RslA complex. Temperature was scanned between 10 °C and 90 °C, with a d*T*/d*t* of 1 °C/min. (d) Zn binding does not confer significant resistance to RslA proteolysis. In this experiment, holo-RslA and apo-RslA were incubated with trypsin (1:2000) for various time points and analyzed on 12% SDS-PAGE. Lane 1, 0 min; lane 2, 5 min; lane 3, 10 min; lane 4, 20 min; lane 5, 30 min; lane 6, 60 min.

A chromophoric Zn^2+^-specific chelator, 4-(2-pyridylazo)-resorcinol (PAR), was used to monitor and quantify the release of Zn^2+^ from RslA and the σ^L^/RslA complex. This assay was performed as described in Materials and Methods (procedure adapted from Bae *et al.*^12^). The kinetics of Zn^2+^ release was monitored after the addition of 10 mM H_2_O_2_. Bound Zn^2+^ is released with a *t*_1/2_ of ∼ 3.5 min. This is substantially slower than *Sco* RsrA, for which the corresponding *t*_1/2_ is ∼ 1.8 min.^12^ The presence of σ^L^ also substantially alters the rate of Zn^2+^ release. The release of bound Zn^2+^ in the σ^L^/RslA complex (*t*_1/2_ ∼ 7 min) is far slower than that of RslA alone (*t*_1/2_ ∼ 3.5 min) (Fig. 2a). The rate of Zn^2+^ release does not significantly differ between the σ_4_^L^/RslA complex and the σ^L^/RslA complex. RslA shows substantial changes in its secondary structural composition upon Zn^2+^ binding. The conformational differences between apo-RslA and holo-RslA were monitored by far-UV circular dichroism (CD) spectroscopy (Fig. 3a). CD spectra of a titration with Zn^2+^ suggested an increase in the α-helical content of RslA with increasing Zn^2+^ concentration. When the Zn^2+^ concentration reached stoichiometric concentrations, no further change in the spectrum of RslA was observed (Fig. 3a). Monitoring the release of Zn^2+^ by CD measurements (mean residue ellipticity at 222 nm) reveals that the conformational change in the protein occurs at a rate substantially slower than that of Zn^2+^ release. The *t*_1/2_ for the conformational change upon Zn^2+^ removal is ∼ 7 min for RslA alone, while that for the σ^L^/RslA complex is ∼ 12 min (Fig. 2b). The differences between the rates of Zn^2+^ release and conformational change thus suggest that while Zn^2+^ release is a rapid response, structural changes provide a much slower second step of the redox-sensing mechanism in RslA. Reduced and oxidized apo-RslA did not show significant structural changes in CD experiments. It is worth noting in this context that while structural rearrangements are more due to Zn^2+^ binding in RslA, these changes in *Sco* RsrA (the other redox-sensitive ZAS protein) are mediated by disulfide bond formation upon oxidation.^23^

The partial unfolding of RslA under oxidizing conditions is also consistent with fluorescent spectroscopy measurements (Fig. 3b). RslA has one Trp residue located in helix H1. The fluorescence spectrum of apo-RslA has an emission maximum at ∼ 354 nm that shifts to ∼ 349 nm upon the addition of Zn^2+^ under reducing conditions. This change in the spectrum suggests that helix H1 undergoes a significant structural rearrangement upon Zn^2+^ binding. The preference for Zn^2+^ as a metal cofactor was noted by changes in the CD spectra when RslA was incubated with other metal ions. Among the other metal ions that were analyzed (viz. Mg^2+^, Mn^2+^, Cu^2+^, Fe^2+^, Ni^2+^, and Co^2+^), none, with the exception of Co^2+^, could replicate the CD spectrum of Zn^2+^-bound RslA (data not shown). Even this was only possible when Co^2+^ was present at a 10-fold molar excess concentration as compared to Zn^2+^.

### Zn^2+^ removal results in a reduced affinity of RslA for σ^L^

An observation in support of the hypothesis that bound Zn^2+^ in RslA plays a functional role is that Zn^2+^ binding substantially influences the ability of RslA to bind σ^L^. The interaction between σ^L^ and RslA was examined by surface plasmon resonance (SPR) measurements. An analysis of the SPR data suggests that the σ^L^/RslA complex has a dissociation constant *K*_d_ of ca 20 nM (Fig. 2c). This affinity is comparable to that reported for the *Sco* σ^R^/RsrA complex (ca 10 nM).^11^ Zn^2+^-free RslA binds σ^L^ with an ∼ 8-fold reduced affinity (*K*_d_ ∼ 150 nM), again comparable to the ca 9-fold reduction observed under similar conditions for *Sco* σ^R^/RsrA (Fig. 2d). The SPR experiments carried out in the presence or in the absence of DTT did not substantially alter the affinity of apo-RslA for σ^L^ (data not shown), suggesting that loss of Zn^2+^ alone (and not disulfide bond formation) plays a role in the interaction of RslA with σ^L^.

### Zn^2+^ binding enhances the structural stability of RslA

Zn^2+^ binding also confers thermostability to RslA. RslA is a stable protein with a *T*_agg_ of 70 °C. Zn^2+^ binding appears to confer thermostability to this protein as apo-RslA has a *T*_agg_ of 60 °C. Binding of RslA also enhances the stability of the σ^L^/RslA complex. Free σ^L^ has a *T*_agg_ of 56 °C, whereas upon RslA binding, the *T*_agg_ of the σ^L^/RslA complex increases to 61 °C (Fig. 3c). Zn^2+^ binding, however, does not confer significant resistance to RslA proteolysis, as both holo-RslA and apo-RslA show similar profiles in a controlled proteolysis experiment using trypsin (Fig. 3d). This finding is different from that of *Sco* RsrA, where Zn^2+^ binding provides significant protection to trypsin digestion.^12^

### Proximal cysteines of the HXXXCXXC motif form a disulfide bond under oxidizing conditions

**Supplementary Fig. 4:**
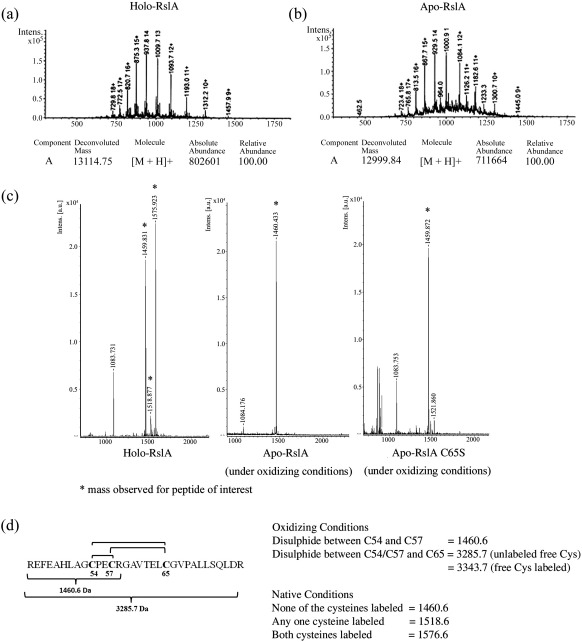
Oxidation involves the formation of a disulfide bond in RslA. (a) LC-ESI-MS spectrum of holo-RslA. (b) LC-ESI-MS spectrum of apo-RslA. (c) Mass spectra of peptides containing the zinc-coordinating residues of RslA. RslA samples were subjected to iodoacetamide labeling, followed by trypsin digestion, to identify the cysteines involved in the disulfide link under oxidizing conditions. (d) Theoretical trypsin digestion profile and probable disulfide connectivity in the region of RslA with three cysteine residues. A list of masses expected for various peptides under oxidizing and native conditions after iodoacetamide treatment is given at the right-hand side of (d).

In the crystal structure of the σ_4_^L^/RslA complex, Zn^2+^ is coordinated by His25, His50, Cys54, and Cys57. The last three residues are from the conserved HXXXCXXC motif. This coordination geometry is similar to that reported for *Sco* RsrA and seen in the crystal structure of *Rsp* ChrR (Fig. 1b). Based on iodoacetamide labeling of cysteines in holo-RslA and apo-RslA and on subsequent analysis by liquid chromatography–electrospray ionization–mass spectrometry (LC-ESI-MS), we note that oxidized RslA has one disulfide bond, whereas there are three free cysteines in holo-RslA. The expected mass for RslA with all three cysteines modified with iodoacetamide is 13,116.5 Da (Supplementary Fig. 4a). The experimentally determined mass for iodoacetamide-treated holo-RslA is 13,114.75 Da, which is within 2 Da of the calculated mass. The oxidized disulfide-bonded RslA with one modified Cys residue has a theoretical mass of 13,000.39 Da that also compares well with the experimentally determined mass of 12,999.84 Da (Supplementary Fig. 4b). In an effort to determine the identity of the cysteines that form the disulfide bond, cysteines in apo-RslA and holo-RslA were modified using iodoacetamide and subsequently proteolyzed using trypsin. Analysis of matrix-assisted laser desorption/ionization time-of-flight (MALDI-TOF) spectra corresponding to these samples revealed that Cys54 and Cys57 form a disulfide in oxidized apo-RslA (Supplementary Fig. 4c). That the distal Cys does not participate in Zn^2+^ binding was verified by characterizing the C65S variant of RslA. The C65S mutant has biochemical properties identical to those of wild-type (wt) RslA. The C54S mutant of RslA, on the other hand, does not bind Zn^2+^ and has a similar far-UV CD spectrum as Zn^2+^-free RslA. The spectroscopic features of the C54S mutant thus reaffirm the hypothesis that structural rearrangements are a consequence of Zn^2+^ removal and are not due to the disulfide bond. RslA thus differs from *Sco* RsrA, where the disulfide involves the distal cysteines (Cys11 and C44) rather than those in the HXXXCXXC motif, and formation of multiple disulfide bonds results in major structural rearrangements.^13^ Indeed, a single disulfide bond in *Sco* RsrA was shown to abolish binding to σ^R^.^23^

### Significance of the XX dipeptide in the CXXC motif

**Supplementary Fig. 5:**
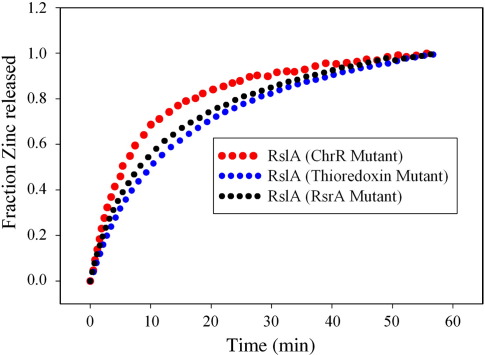
Zn^2+^ release kinetics for the XX dipeptide mutants of RslA.

ZAS proteins show substantial variations in their redox sensitivity. The Zn^2+^ cofactor in the ASD of *Rsp* ChrR was suggested to play a more structural role rather than to serve as a sensor for singlet oxygen or other oxidative stimuli. To evaluate if these differences could be explained by the sequence of the XX dipeptide in the CXXC motif, we examined mutants of RslA with different dipeptide sequences. In this experiment, the CXXC motif of RslA (CPEC) was mutated to CDEC, CSPC, and CGPC, corresponding to those of *Rsp* ChrR, *Sco* RsrA, and *Ec* thioredoxin, respectively. These mutants of RslA were purified using a procedure similar to that used in wt protein, and the kinetics of Zn^2+^ release were examined. We note that all dipeptide mutants release Zn^2+^ in the presence of H_2_O_2_. The *t*_1/2_ values for Zn^2+^ release vary, however, ranging from ∼ 6.5 min (*Rsp* ChrR variant), to ∼ 13 min (*Ec* thioredoxin), to ∼ 15 min (*Sco* RsrA), all higher than that observed for wt-RslA (∼ 3.5 min) (Fig. 3; Supplementary Fig. 5).

### Interactions of the σ^L^ domains with RslA

**Supplementary Fig. 6:**
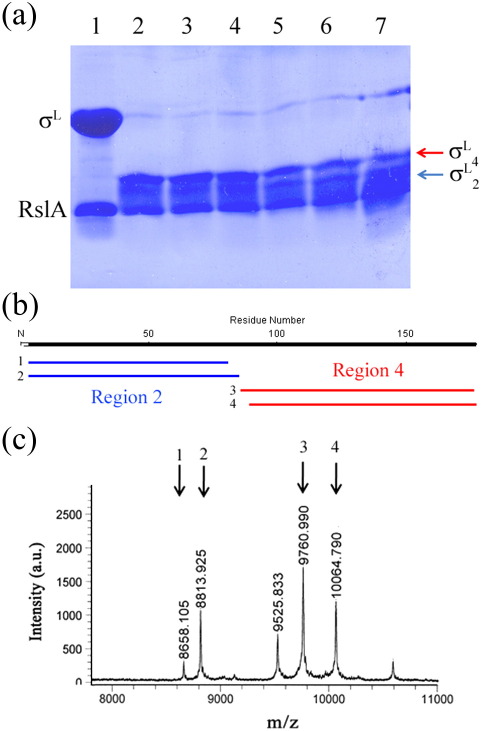
(a) Limited proteolysis of the σ^L^/RslA complex. σ^L^ binding to RslA confers protection from proteolysis. The complex was incubated with trypsin (1:2000) for various time points and analyzed on 12% SDS-PAGE. Lane 1, 0 min; lane 2, 5 min; lane 3, 10 min; lane 4, 20 min; lane 5, 30 min; lane 6, 60 min. (b) σ_2_^L^ and σ_4_^L^ were mapped onto the σ^L^ sequence. These data suggest that σ^L^ binding makes the ASD of RslA resistant to proteolysis. It therefore appears likely that proteolytic removal of the ASD may not be a mechanism to release free σ^L^ from the σ^L^/RslA complex. (c) Representative MALDI-TOF spectrum of the proteolyzed sample. MALDI-TOF peaks marked in the spectrum correspond to the σ_2_^L^ or σ_4_^L^ domains of σ^L^ (top).

**Supplementary Fig. 7:**
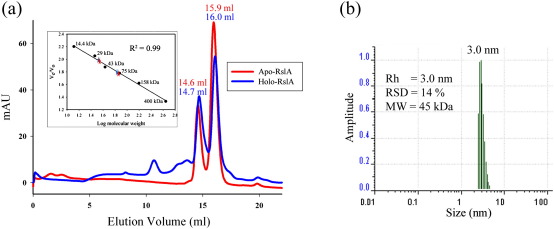
(a) Analytical gel-filtration profile of holo-RslA and apo-RslA suggests that RslA exists as dimer and tetramer in solution. Inset: The calibration curve obtained using standard molecular weight markers. Red and blue stars in the inset correspond to the observed molecular weights of apo-RslA and holo-RslA. (b) DLS experiments suggest a hydrodynamic radius (*R*_h_) of 3.0 nm with a high relative standard deviation of ~ 14%. The dimeric and tetrameric oligomers of RslA could not be resolved by DLS experiments.

A limited proteolysis experiment with the σ^L^/RslA complex reveals that σ^L^ is cleaved into two stable domains (σ_2_^L^ and σ_4_^L^) within a minute of initiating the proteolysis reaction (Supplementary Fig. 6). This is consistent with observations from crystallization trials on the intact σ^L^/RslA complex. However, we note that RslA is protected from proteolysis upon forming a complex with σ^L^. RslA alone, on the other hand, is very susceptible to proteolysis. In an effort to identify the relative contributions of the two domains of σ^L^ to complex formation, we monitored the interactions of the two domains with RslA by SPR. To our surprise, σ_4_^L^ alone did not interact with RslA. Homooligomerization of this domain appears to be the likely cause for this observation (size-exclusion chromatography; data not shown). σ_2_^L^, on the other hand, binds RslA, albeit with an ∼ 100-fold reduction in its affinity as compared to intact σ^L^ (*K*_d_∼ 2 μM). However, the results of protein–protein interaction experiments using coexpression and copurification were more conclusive. Of the two constructs in the pETDuet-1 vector having σ_2_^L^ and σ_4_^L^ in multiple cloning site 1 (Supplementary Table 2, constructs 5 and 6) with a poly-histidine tag at the N-terminus and RslA in multiple cloning site 2, RslA copurified with σ_4_^L^, whereas the σ_2_^L^/RslA complex could not be obtained. We also note that both apo-RslA and holo-RslA (Supplementary Table 2, construct 7) self-associate into dimeric and tetrameric oligomers in solution. This feature is seen in analytical gel-filtration experiments and is consistent with data from dynamic light scattering (DLS) measurements (Supplementary Fig. 7). A small shift in elution profile was observed in analytical gel-filtration experiments with the apo and holo forms, suggesting that Zn^2+^-bound RslA adopts a more compact conformation. The self-association of RslA in solution appears similar to the phage-λ-encoded protein AsiA that is also predominantly a dimer in solution. As in the case of σ^L^/RslA and the σ_4_^L^/RslA complex, AsiA forms a 1:1 complex with the *Ec* primary σ factor σ^70^.^24^

## Discussion

ECF σ factors respond to a variety of environmental signals that include periplasmic stress in Gram-negative bacteria, oxidative or disulfide stress, changes in light intensity, or resistance to cobalt or nickel.^2,25,26^ The receptor for these diverse signals is often located in the anti-σ factor that responds by releasing the σ factor upon specific environmental cues. *Mtb* RslA is a ZAS protein with a cytosolic domain (ASD) that interacts with the ECF σ factor σ^L^ and a membrane-spanning C-terminal domain. Of the two promoter recognition domains σ_2_ and σ_4_, σ_4_ is often a target for various regulators. Examples of this feature include λcI, Rhas, CRP, MotA, and anti-σ factors.^27^ An analysis of the structures of some of these σ factor/anti-σ complexes, notably *Ec* RseA, *Rsp* ChrR, *Salmonella typhimurium* FlgM, and *Ec* Rsd, suggests that these proteins inactivate their cognate σ factors by occluding both the RNAP binding sites and the promoter binding regions.^4,28–30^ More importantly, *Ec* AsiA and *Rsp* ChrR also bring about structural rearrangements in σ_4_ that affect its promoter binding ability.^3,18^ In the *Ec* FecI/FecR σ/anti-σ factor pair, σ_4_ plays a dominant role in interactions, as in the case of *Ec* AsiA and Rsd, which interact with σ_4_^A^ with similar affinities.^31,32^ The distinct mechanisms of interactions in these two anti-σ factors were reported to have functional implications, with AsiA being a more potent inhibitor of transcription.^32^ The *Mtb* σ^L^/RslA complex differs from both the *Ec* σ^E^/RseA complex and the *Rsp* σ^E^/ChrR complex, where the σ_2_ and σ_4_ domains contribute similarly to the σ/anti-σ complex. *Ec* RseA, for example, was shown to pull down both domains of *Ec* σ^E^.^4^ Instead, the σ^L^/RslA interactions appear closer to that seen in the *Sco* σ^R^/RsrA or *Ec* FecI/FecR systems, where one of the promoter binding domains interacts with the anti-σ factor with a significantly higher affinity than the other.^31,33^

**Fig. 4:**
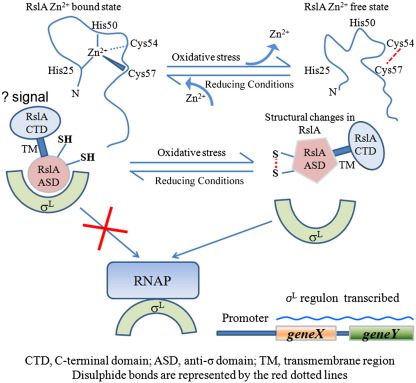
Schematic of the mechanism by which RslA regulates σ^L^. Please refer to the text of the article for details.

*Mtb* RslA and *Rsp* ChrR are the only ZAS proteins for which structures have been determined. Of these, *Rsp* ChrR has two domains: the ASD and a double-stranded β-helix (cupin) domain. In this case, ASD is involved in binding σ^E^, while the cupin domain serves as the sensor of singlet oxygen levels.^3^ The other characterized ZAS proteins to date include *Mtb* RshA and *Sco* RsrA, both of which are sensitive to redox stimuli.^23,34^ RslA responds to oxidative stimuli with the release of the Zn^2+^ cofactor, followed by the formation of a disulfide link between proximal cysteines of the HXXXCXXC motif (Fig. 4). While this disulfide link differs from the other characterized ZAS protein *Sco* RsrA, it is closer to redox sensor proteins such as Spx, ResA, and Hsp33.^35–37^ Although there appeared to be some discrepancy in the reports on Zn^2+^ coordination and disulfide connectivity in *Sco* RsrA, it appears likely that all the three conserved residues in the HXXXCXXC motif are involved in Zn^2+^ binding.^12,13,23^ The Zn^2+^ coordination geometry is different from *Sco* RsrA in *Rsp* ChrR, as the distal Cys11 residue is replaced by a histidine.^3^ *Rsp* ChrR does not respond to oxidative stress; instead, it acts as a sensor for singlet oxygen species, with this role performed by a different domain.^3^ Two probable reasons were cited for the insensitivity of ChrR to oxidative stress: (i) surface inaccessibility of Cys, or (ii) the chemical nature of the Zn^2+^ ligands. Both Cys residues in the conserved CXXC motif of *Mtb* RslA are solvent exposed, unlike *Rsp* ChrR, where only Cys35 is surface accessible while Cys38 is not (Supplementary Fig. 3c and d). This observation is analogous to the Zn^2+^ binding redox sensor of Hsp33 where three of four Cys residues that coordinate Zn^2+^ are accessible (PDB ID 1VQ0). The sequence of the XX dipeptide in the CXXC motif has been shown to be crucial in controlling the redox properties of the protein in which it is found.^36,38,39^ In the present analysis, the XX dipeptide in the CXXC motif of RslA was mutated to the corresponding sequences of the other characterized ZAS proteins *Sco* RsrA and *Rsp* ChrR, and of a thioldisulfide oxidoreductase (*Ec* thioredoxin). In this study, we examined the rate of release of bound Zn^2+^ with an oxidative stimulus. All the RslA dipeptide variants showed an increase in *t*_1/2_ for Zn^2+^ release when compared to wt-RslA. It thus appears likely that the redox sensitivity in ZAS proteins involves other sequence structure features apart from the dipeptide sequence in the CXXC motif.

In summary, the crystal structure of the σ_4_^L^/RslA complex reveals that RslA inactivates σ^L^ by sterically occluding both the promoter and the RNAP binding regions on σ_4_^L^. The structure also provides a rationale for the biochemical characteristics of this ZAS protein. These results thus provide a template for understanding the mechanism by which RslA ASD regulates σ^L^, while providing a model for further studies to understand the sequence structure features of ZAS proteins that contribute to redox sensitivity.

## Materials and Methods

### Cloning, expression, and purification of different σ^L^ and anti-σ^L^ constructs

The details of the expression constructs used in this study are compiled in Supplementary Table 2. Site-directed mutagenesis was carried out, using a single primer-based PCR method, to obtain point variants of RslA.^40^ The σ_4_^L^/RslA and σ^L^/RslA complexes were purified with a coexpression and copurification strategy using pETDuet-1 (Novagen, Inc.) expression vector. All protein samples were purified using the same protocol. Briefly, after the plasmid had been transformed into Rosetta(DE3)pLysS cells (Novagen, Inc.), the cells were grown in Luria broth with antibiotics (100 μg/ml ampicillin and 30 μg/ml chloramphenicol) to an *A*_600_ of 0.5–0.6. The cells were induced with 0.5 mM IPTG. Subsequently, growth temperature was lowered to 290 K, and the cells were grown for 12–18 h before they were spun down. Cells were lyzed by sonication in lysis buffer [20 mM Tris (pH 7.5) and 250 mM NaCl]. Cell-free lysate was incubated with Co-NTA beads and eluted by a gradient of elution buffer [20 mM Tris–HCl (pH 7.5), 250 mM NaCl, and 200 mM imidazole]. Recombinant proteins were further purified by size-exclusion chromatography using a Superdex S-200 column (GE Healthcare) after the affinity chromatography step. For analytical gel-filtration experiments, ∼ 0.2-mg to 0.5-mg protein samples were passed through a Superdex S-200 10/300 GL column equilibrated in 20 mM Tris–HCl (pH 7.5) and 200 mM NaCl at 6 °C. The proteins were then concentrated using a membrane-based centrifugal ultrafiltration system (Amicon-Ultra). The molecular weights of the recombinant proteins were verified by MS on both MALDI-TOF and LC-ESI-MS (Bruker Daltonics, Inc.).

### Crystallization and structure determination of σ_4_^L^/RslA

Initial crystallization conditions were obtained by hanging-drop vapor diffusion in 24-well plates using a sparse matrix screen (Hampton Research) at room temperature by mixing 2 μl of protein solution with 2 μl of well solution and equilibrating against 400 μl of well solution. Diffraction-quality crystals for the σ_4_^L^/RslA complex were obtained by combining 2 μl of protein solution (∼ 10 mg/ml) and 2 μl of well solution [0.1 M 2,2'-(Propane-1,3-diyl iimino)bis[2-(hydroxymethyl)propane-1,3-diol] propane (pH 7.0), 0.8–1.2 M ammonium sulfate, and 0.8–5% polyethylene glycol 8000] at room temperature. Crystals appeared within ∼ 2 days of tray setup and grew to maximum dimensions in 1–2 weeks. The crystals were flash-frozen in a cryoprotectant solution containing 15% glycerol. Diffraction data were collected at wavelengths of 1.282 Å and 0.9762 Å at beamline BM14 of the European Synchrotron Radiation Facility. The data were processed using iMOSFLM^41^ and scaled using SCALA.^42^ Zn^2+^ substructure was located using PHENIX.^43^ Ten Zn^2+^ sites were located in the asymmetric unit, with each site corresponding to one molecule of the complex. Subsequent refinement and manual model building were performed using REFMAC^44^ and Coot.^45^ TLS refinement was performed using REFMAC.^44^ The final model contains 1661 residues, 10 Zn^2+^, 10 SO_4_^2−^, and 269 water molecules. Analysis of the structure showed 97.8% of the residues in the most favored regions of the Ramachandran plot and 2.2% of the residues in additionally allowed regions (no residues in generously allowed or disallowed regions). Crystals of the σ^L^/RslA complex (protein concentration, ∼ 7 mg/ml) were obtained after a year of setting up the hanging-drop experiment in 0.5 M succinic acid (pH 7.0) and 0.1 M 2,2'-(Propane-1,3-diyl iimino)bis[2-(hydroxymethyl)propane-1,3-diol] (pH 7.0). Upon SDS-PAGE and LC-ESI-MS analysis, we could deduce that σ^L^ was proteolyzed with the removal of σ_2_^L^. One of these crystals diffracted to 3.2 Å resolution at the home source, and the structure was solved by molecular replacement using the σ_4_^L^/RslA complex as search model in PHASER.^46^ No electron density was observed for σ_2_^L^ domain in this structure.

### Preparation of apo-RslA and zinc-saturated RslA

Apo-RslA was prepared by oxidizing the sample with 1 mM H_2_O_2_ in the presence of 5 mM *N*,*N*,*N*′,*N*′-tetrakis(2-pyridylmethyl) ethylenediamine (TPEN) (a Zn^2+^ chelator). Excess TPEN and H_2_O_2_ were subsequently removed by gel-filtration chromatography. To prepare Zn^2+^-saturated RslA, we incubated purified RslA with ZnCl_2_ under reducing conditions overnight at 4 °C. The unbound Zn^2+^ was removed by several washes of buffer using a centrifugal ultrafiltration system (Amicon-Ultra).

### Fluorescence spectroscopy

All fluorescence studies were performed on a JOBIN YVON FlouroMax-3 fluorimeter at room temperature. Fluorescence excitation was set to 280 nm, and emission spectra were recorded from 300 nm to 400 nm at a bandwidth of 1 nm. The excitation and emission slit widths were set to 3 mm and 5 mm, respectively. Each recorded spectrum represents an average of five scans. The spectra were obtained at a protein concentration of 5–10 μM in 5 mM Tris–HCl (pH 7.5) in the presence of 1 mM DTT.

### CD spectroscopy

CD experiments were carried out on a JASCO-J715 instrument. All spectra were recorded in 5 mM Tris–HCl buffer adjusted to pH 7.5, with 1 mM DTT, using a 1-mm pathlength cuvette. Protein concentrations were kept in the range of 5–15 μM. Each reported spectrum is an average of five scans.

### PAR assay for zinc measurements

The metal chelator PAR, when complexed with free Zn^2+^, absorbs light intensively at 500 nm.^12^ The time course of Zn^2+^ release under oxidizing and reducing conditions was measured. Briefly, Zn^2+^-saturated samples were prepared as described earlier. For the assay, protein samples were diluted to 5–10 μM in a buffer containing 0.1 mM PAR. All buffers were purged with N_2_ gas. Nonspecifically bound Zn^2+^ was trapped by PAR in the absence of oxidation, as monitored by a slight increase in absorbance at 500 nm. When the absorbance had stabilized, 10 mM H_2_O_2_ was added, and *A*_500_ was monitored every 30 s for 25–50 min at 25 °C.

### Limited proteolysis experiments

Limited proteolysis experiments were carried out at room temperature with 1:2000 protease/protein concentration. The samples were incubated for various time points. For MALDI-TOF experiments, reactions were stopped by adding 1% trifluoroacetic acid (final concentration). For SDS-PAGE, analysis reactions were stopped by adding the loading dye solution and by boiling the samples for 5 min.

### SPR measurements

The kinetics of interaction between RslA and σ^L^ were examined using SPR (BIACORE 2000; GE Healthcare). RslA was immobilized on a CM5 chip (BIACORE; GE Healthcare) at a surface density of 12 ng/mm.^2^ σ^L^, σ_2_^L^, and σ_4_^L^ at varying concentrations were used as analytes. The first panel of the CM5 chip served as control. Interaction studies were carried out in 20 mM Tris–HCl buffer (pH 7.8), 200 mM NaCl, and 50 mM imidazole. For experiments with apo-RslA, a buffer containing 10 mM H_2_O_2_ and 2 mM TPEN was passed on the chip to remove bound Zn^2+^. A reducing environment was maintained with 1 mM DTT for interaction studies with holo-RslA, although no changes were seen in SPR chromatograms without DTT. For interaction studies with apo-RslA, a buffer without DTT was used. Binding constants were estimated using the BIA-evaluation software (BIACORE; GE Healthcare).

### LC-ESI-MS and MALDI analysis of RslA

The cysteines in apo-RslA and holo-RslA were modified using iodoacetamide, which provides a mass difference of 58 Da for each modified Cys residue. Both apo-RslA and holo-RslA samples were incubated with an ∼ 50-fold molar excess of iodoacetamide in 50 mM ammonium bicarbonate (pH 8.0) at 37 °C for 45 min. LC-ESI-MS (Bruker Daltonics, Inc.) was carried out using a C8 analytical column. The differences in mass were within the error range of ± 2Da. For MALDI-TOF MS experiments (Bruker Daltonics, Inc.), the samples were proteolyzed using a 1:2000 trypsin/protein ratio and subsequently desalted using a 1-ml Superdex G-25 column.

### Thermal denaturation experiments

Thermal denaturation experiments were carried out on a UV–visible spectrophotometer (JASCO V-530) at a fixed wavelength of 400 nm in a 10-mm pathlength quartz cell. The protein concentrations used varied from 5 μM to 10 μM in 250 mM NaCl and 20 mM sodium phosphate buffer (pH 7.5). The temperature was varied from 10 °C to 90 °C at a rate of 1 °C/min.

### Analytical size-exclusion chromatography

For analytical gel-filtration experiments, ∼ 0.2-mg to 3-mg protein samples were passed through a Superdex S-200 10/300 GL column equilibrated in 20 mM Tris–HCl (pH 7.5) and 200 mM NaCl at 6 °C. The flow rate was maintained at 0.3 ml/min.

### DLS experiments

DLS experiments were performed using Viscotek 802 DLS in a quartz cuvette (volume, 12 μl) at 22 °C. Protein concentrations were kept in the range of about ∼ 1 mg/ml.

### Accession number

Coordinates and structure factors have been deposited in the PDB with PDB ID 3HUG.

## Figures and Tables

**Supplementary Fig. 8:**
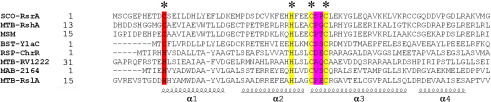
Sequence comparison of representative ZAS proteins. *Sco* RsrA, *Streptomyces coelicolor* RsrA; *Mtb* RshA, *Mycobacterium tuberculosis* RshA; *bst*ylac,*Bacillus subtilis*Ylac σ factor; MSM, anti-σ factor from *Mycobacterium smegmatis* str. MC;^2^*Rsp* ChrR, *Rhodobacter sphaeroides* ChrR; *Mtb* RV1222, *Mycobacterium tuberculosis* anti-σ^E^, *Mycobacterium abscessus* hypothetical protein MAB_2164; *Mtb* RslA, *Mycobacterium tuberculosis* RslA. The asterisk on the top of sequence alignment represents probable residues involved in zinc binding. The residues shown against a red background are involved in Zn^2+^ coordination (either cysteine or histidine). The conserved zinc binding motif residues are shaded yellow. The sequences of XX dipeptide in conserved CXXC motif in anti-σ factors are shown in pink. The secondary structural elements shown are based on the crystal structure of RslA (PDB ID 3HUG).

## Appendix A Supplementary data

Supplementary Table 1 Data collection, phasing, and refinement statistics.

Supplementary Table 2 Expression constructs of σ^L^ and RslA used in this study.
