# Supplementary material for: Structural and Biochemical Bases for the Redox Sensitivity of Mycobacterium tuberculosis RslA
Source: J Mol Biol. 2010 Apr 16;397(5):1199–208. doi: 10.1016/j.jmb.2010.02.026 (PMC2877774; doi:10.1016/j.jmb.2010.02.026)
Supplement: Supplementary Table 1 — Data collection, phasing, and refinement statistics. [file mmc1.doc]

**Table 1: Data Collection, Phasing and Refinement statistics**

| **Data Collection** | | |
| --- | --- | --- |
|  | **σ^L^_4_/RslA** (Zn-Peak) | **σ ^L^_4_/RslA** |
| Wavelength | 1.2821 Å | 0.9762 Å |
| Resolution (Å) | 49.12-2.7 (2.85-2.7) ^a^ | 44.72-2.35 (2.48-2.35) |
| Unit cell parameters | a =83.05 Å, b = 166.79 Å,  c = 178.44 Å | a =83.07 Å, b = 166.77 Å,  c = 178.87 Å |
| Space group | P2_1_2_1_2_1_ | P2_1_2_1_2_1_ |
| Completeness (%) | 100.0 (100.0) | 99.7 (100.0) |
| Multiplicity | 10.3 (10.3) | 4.2 (4.1) |
| R_merge_ (%) ^b^ | 8.4 (48.3) | 6.5 (48.1) |
| <I>/σ(I) | 16.4 (4.4) | 12.4 (3.0) |
| **Refinement Statistics** | | |
| Total no. of reflections used | 65355 | 98462 |
| R_cryst_ ^c^ /R_free_ ^d^ (%) | 25.4/27.6 | 21.5/25.6 |
| rmsd_bond_ (Å) | 0.010 | 0.011 |
| rmsd_angle_ (deg.) | 1.36 | 1.40 |
| **No. of Atoms (Non-Hydrogen)** | | |
| Protein | 9891 | 10447 |
| Water | 238 | 269 |
| Zinc | 10 | 10 |
| Sulphate | - | 10 |
| **Mean B factors (Å^2^)** | | |
| Overall | 55.78 | 46.42 |
| Protein | 56.17 | 43.18 |
| Water | 43.55 | 51.11 |
| Zinc | 52.46 | 42.49 |
| **Ramachandran Plot Statistics** | | |
| Residues in most favoured regions (%) | | 97.8 |
| Residues in additional allowed region (%) | | 2.2 |
| Residues in the disallowed region (%) | | 0 |

^a^Values for highest resolution bin are given in parenthesis.

^b^R_merge_=Σ_j_|<I>-I_j_|/Σ<I> where I_j_ is the intensity of the j^th^ reflection and <I> is the average intensity.

^c^R_cryst_ = Σ||F_observed_| − |F_calculated_||/Σ|F_observed_|

^d^R_free_ = R_cryst_ calculated with 5% data randomly omitted from the refinement.

**Table 2: Expression constructs of σ^L^ and RslA used in this study**

| **S.No.** | **Vector** | **MCS1** | **MCS2** | **Solubility** |
| --- | --- | --- | --- | --- |
| 1 | pET-Duet1 | N-term His-tag σ^L^ _(1-177)_ | RslA _1-108_ | ~7mg/ml |
| 2 | pET-Duet1 | N-term His-tag σ^L^ _(1-177)_ | – | ~0.3mg/ml |
| 3 | pET-Duet1 | N-term His-tag σ^L^_2 (1-95)_ | – | ~1mg/ml |
| 4 | pET-Duet1 | N-term His-tag σ^L^_4 (100-177)_ | – | ~5mg/ml |
| 5 | pET-Duet1 | N-term His-tag σ^L^_2 (1-95)_ | RslA _1-108_ | ~1mg/ml |
| 6 | pET-Duet1 | N-term His-tag σ^L^_4 (100-177)_ | RslA _1-108_ | ~15mg/ml |
| 7 | pET-Duet1 | N-term His-tag RslA _(1-108)_ | – | ~25mg/ml |
| 8 | pET-Duet1 | N-term His-tag RslA _(1-108)_ C54S | – | ~25mg/ml |
| 9 | pET-Duet1 | N-term His-tag RslA _(1-108)_ C65S | – | ~25mg/ml |
| 10 | pET-Duet1 | N-term His-tag RslA _(1-108)_P55D  (Rsp ChrR ‘XX’ mutant) | – | ~25mg/ml |
| 11 | pET-Duet1 | N-term His-tag RslA _(1-108)_ P55S; E56P (Sco RsrA ‘XX’ mutant) | – | ~25mg/ml |
| 12 | pET-Duet1 | N-term His-tag RslA _(1-108)_ P55G;E56P (Ec Thioredoxin ‘XX’ mutant) | – | ~25mg/ml |

**Foot-note**: pETDuet-1: *E. coli* expression vector (Novagen, Inc.), MCS: Multiple Cloning Site
